# Patients with low back pain presenting for chiropractic care who want diagnostic imaging are more likely to receive referral for imaging: a cross-sectional study

**DOI:** 10.1186/s12998-022-00425-5

**Published:** 2022-04-04

**Authors:** Hazel J. Jenkins, Alice Kongsted, Simon D. French, Tue Secher Jensen, Klaus Doktor, Jan Hartvigsen, Mark Hancock

**Affiliations:** 1 Faculty of Medicine, Health and Human Sciences, Macquarie University, Sydney, Australia; 2 Department of Sports Science and Clinical Biomechanics, University of Southern Denmark, Odense, Denmark; 3 Chiropractic Knowledge Hub, Odense, Denmark; 4 Silkeborg Regional Hospital, Silkeborg, Denmark

**Keywords:** Diagnostic imaging, Low back pain, Chiropractic, Patient beliefs

## Abstract

**Background:**

It is unclear if the use of imaging for low back pain (LBP) is impacted by patient beliefs. This study aimed to: (1) describe beliefs about the importance of imaging and whether patients wanted imaging when presenting for chiropractic care for LBP; (2) describe associations between baseline patient characteristics and imaging beliefs and whether patients wanted imaging; and (3) determine whether patients who believed imaging to be important in the management of LBP, or who wanted to receive imaging, were more likely to receive an imaging referral.

**Methods:**

Cross-sectional observational data was collected between November 2016 to December 2019 from 10 primary care chiropractic clinics in Denmark. Consecutive patients aged 18 or older and presenting with a new episode of LBP were included (N = 2818). Beliefs about the importance of imaging (two questions) and whether imaging was wanted (one question) were collected at the initial visit, together with baseline participant characteristics and whether an imaging referral was provided. Associations between imaging beliefs/desire to receive imaging and participant characteristics were explored using multivariable logistic regression analysis. The relationships between imaging beliefs and desire to receive imaging with subsequent imaging referral were assessed using multivariable logistic regression analysis adjusted for pre-selected confounder variables.

**Results:**

Approximately one third of participants believed imaging to be important for the management of LBP (29.5% (95%CI 27.8, 31.3) or 41.5% (95%CI 39.6, 43.3) depending on the two imaging beliefs questions). Approximately one quarter (26.1%, 95%CI 24.5, 27.7) of participants wanted to receive an imaging referral. Participants were more likely to believe in the importance of imaging or want an imaging referral if they had a longer duration of LBP, history of previous imaging for LBP, or a lower completed education level. Participants who wanted imaging at the initial consult were more likely to receive an imaging referral (Odds ratio; 95%CI 1.6; 1.2, 2.1).

**Conclusions:**

Approximately one third of patients presenting for chiropractic care in Denmark believed imaging to be important in the management of LBP. One quarter wanted imaging at the initial consult. Patients’ desire for imaging appeared to impact the use of diagnostic imaging.

**Supplementary Information:**

The online version contains supplementary material available at 10.1186/s12998-022-00425-5.

## Background

Imaging is commonly used in the management of low back pain (LBP) [1] and approximately one third of imaging is considered to be referred for inappropriately when compared to recommendations in clinical practice guidelines [2]. The routine use of imaging in the management of LBP is discouraged due to limited evidence of patient benefit and increased risk of harm from overdiagnosis, unnecessary further investigation or treatment, and radiation exposure. However, attempts to decrease practitioner referrals for low back imaging have been largely unsuccessful [3].

Patient requests for imaging have been suggested by clinicians as a key barrier to reducing inappropriate imaging use in primary care [4]. Previous studies have shown that both patients presenting for medical care [5, 6] and the general population [7, 8] believe imaging to be important in the management of LBP. Patient beliefs that imaging is needed for the management of LBP has also been associated with increased imaging use [9] and increased referral for inappropriate imaging [5]. An intervention educating patients about the need and usefulness of imaging for LBP [10] decreased imaging referrals by general medical practitioners [11], reinforcing that addressing patient beliefs may be needed to help reduce referral for inappropriate imaging.

Imaging is also commonly used for LBP patients presenting for chiropractic treatment [12]. Although identified as a potential barrier to reducing unnecessary imaging [13], chiropractors only rarely identify patient requests for imaging as a reason for deciding to refer for imaging of the low back [14, 15]. It is currently unclear whether patients presenting for chiropractic care believe that imaging is important for the management of LBP. Likewise, it is unknown whether patient beliefs about the importance of imaging or their desire for imaging impact subsequent imaging referral in chiropractic clinical practice. Therefore, it is uncertain whether interventions to increase the appropriate use of imaging use in chiropractic practice should also address patient beliefs.

The aims of this study were to:

1. Describe beliefs in the importance of imaging and whether patients wanted imaging when presenting for chiropractic care for LBP
2. Describe associations between baseline patient characteristics and imaging beliefs and whether patients wanted imaging
3. Determine whether patients who believed imaging to be important in the management of LBP, or who wanted to receive imaging prior to seeing the chiropractor, were more likely to receive an imaging referral independent of participant characteristics, LBP symptom history/severity and the treating chiropractor’s imaging referral habits

## Methods

We used baseline data from the Danish Chiropractic LBP cohort (ChiCo) study to undertake a cross-sectional analysis. This is the second of two analyses we have undertaken, the other is published elsewhere [16]. ChiCo is a prospective longitudinal observational study with one year follow-up performed between November 2016 and December 2019 [17]. As the ChiCo study followed ethical regulations [17] and only anonymised data were provided for use in this study, the Macquarie University Human Research Ethics Committee provided a waiver to use the ChiCo data without further ethical review. The Health Research Committee of Southern Denmark determined that the ChiCo project did not require ethical approval (S-20.162.000-109). Storing and processing of personal data was registered with the Danish Data Protection Agency via the University of Southern Denmark’s joint registration system (2015-57-0008; file # 16/47215). Reporting of this paper is in accordance with the STROBE statement (Additional file 1).

### Cohort

As described elsewhere [16, 17], the ChiCo cohort included 2818 patients who presented to chiropractors in Denmark. To be included, patients needed to: have a new or recurrent episode of LBP, with or without leg pain; be 18 years old or over; and, be able to complete electronic questionnaires in Danish. Patients with a confirmed diagnosis of fracture, infection, cancer, or other serious pathology were excluded, as were patients who were in an ongoing course of treatment or long-term management. When determined to be eligible, participants completed an initial questionnaire before they underwent a baseline clinical assessment with the treating chiropractor, who then completed a clinical assessment questionnaire. Following the assessment, participants completed a second baseline questionnaire. Treatment provided by the chiropractors was as needed and not influenced by participation in the study. No limitation was placed on access to other healthcare services. For the current study, all participants were eligible for inclusion for Aims 1 and 2. For Aim 3, participants who were identified by the chiropractor to have previous imaging relevant to the presenting LBP complaint were excluded from analysis.

### Data collection and outcome measures

Questions regarding imaging beliefs and desire for imaging were collected in the initial baseline questionnaire, completed before the clinical assessment. Participant beliefs regarding the importance of imaging were assessed by two questions used in previously published research [6]. The questions asked participants to rate their agreement on a five-point Likert scale, from strongly disagree to strongly agree, to the statements: (1) ‘X-rays or scans are necessary to get the best medical care for LBP’ and (2) ‘Everyone with LBP should have spine imaging (e.g. X-ray, CT, or MRI)’. Responses were dichotomised for analysis into those that agreed or strongly agreed with the statement compared to all other responses. Participants were then asked “What do you want from your visit with the chiropractor? You can mention things you want even if you are uncertain that they will be part of the visit”. This was followed by a list of items that could be answered “yes” or "no”, one of the items being: ‘Radiography or MRI will be performed or I will be referred for it’. Finally, whether participants were referred for imaging in the initial assessment was collected in the clinical assessment questionnaire. Chiropractors were asked to respond (yes/no) to three items: ‘Will the patient be referred to radiography’; ‘Will the patient be referred to CT’; ‘Will the patient be referred to MRI’. The participant was considered to have been referred for imaging if a response of ‘yes’ was received for any of these three items.

Covariates were assessed in the two participant baseline questionnaires and the clinical assessment questionnaire completed by the chiropractor. The initial baseline questionnaire (completed before the clinical assessment) included: age; sex (male/female); LBP intensity, measured on a 0–10 numerical rating scale as average or typical pain over the previous week; low back disability, measured using the Danish 23-item Roland Morris Disability Questionnaire (RMDQ) with results proportionally recalculated to a score from 0 to 100; duration of current episode of LBP (1–2 days; 3–7 days; 1–2 weeks; 2–4 weeks; 1–3 months; 3–12 months; more than 1 year); previous imaging for back pain (yes/no); and back pain beliefs measured using the Back Beliefs Questionnaire (scored from 9 to 45, lower score indicating more negative beliefs) [18]. The second baseline questionnaire (completed after the clinical assessment) included: education level (eight categories from no education to higher education, more than 4 years); and previous LBP (none, 1 episode, 2–3 episodes, more than 3 episodes). The LBP diagnosis determined by the chiropractor (non-specific LBP, spine-related leg pain with nerve root involvement, spine-related leg pain without nerve root involvement, or suspected fracture, infection, cancer, cauda equina syndrome, inflammatory arthritis) was collected in the clinical assessment questionnaire.

### Data analysis

Multiple imputation was performed using SPSS (IBM Corp. Released 2017. IBM SPSS Statistics for Windows, Version 25.0. Armonk, NY: IBM Corp.) for covariates where there was less than 5% missing data (LBP intensity, LBP duration, back beliefs) and for two items from the second baseline questionnaire which was not completed by 27% of participants (previous LBP, education level). Missing data for outcome or exposure variables, including beliefs regarding the importance of imaging, whether participants wanted imaging, and referral for imaging were not imputed, and participants with missing data were removed from the analysis.

Sample size calculations were performed for Aim 3 as a smaller subset of the ChiCo cohort (2053/2818 participants) was available for analysis due to exclusion of participants with previous relevant imaging and missing outcome data. Sample size calculations were performed in GPower 3.1.9.2. Assuming an imaging frequency of 25% [1] and a high correlation between covariates and the outcome measure, the available sample size would have 80% power to detect an odds ratio of 1.9.

Participant beliefs of the importance of imaging (Aim 1) were presented descriptively as the percentage of respondents for each Likert scale category (strongly disagree to strongly agree) and the dichotomised responses (agree and strongly agree compared to all other responses) with 95% confidence intervals. The number of patients wanting imaging (Aim 1) was presented as a percentage with 95% confidence interval.

Univariable and multivariable logistic regression analyses were performed to assess for association between the pre-selected clinical and demographic factors and participant beliefs of the importance of imaging and expectations for imaging (Aim 2). No additional confounder variables were selected. The nine baseline factors were selected by author consensus and informed by previous research [6], and comprised: age, sex, LBP intensity, low back disability, duration of current episode of LBP, previous imaging for back pain, back beliefs, education level, and previous LBP. Pain duration was dichotomised for analysis into four weeks or less or more than four weeks. Education level was dichotomised into below further education (no education, primary school, youth education, vocational education) or further education and above (short further education, middle further education, higher education). Previous LBP was dichotomised into yes (any episodes of previous LBP) or no. Three models were created, each using the same clinical and demographic baseline factors as independent variables and the dichotomised responses to the two imaging beliefs questions and whether patients wanted imaging as dependent variables respectively. Odds ratios and 95% confidence intervals were calculated for the variables in each model. Multicollinearity of the independent variables was assessed, and any variable with a variance inflation factor (VIF) of three or more was removed from the models.

Whether patients who believed imaging to be important or wanted to receive imaging were more likely to get an imaging referral was investigated using univariable (unadjusted) and multivariable (adjusted for pre-selected potential confounders) logistic regression analysis (Aim 3). The pre-selected confounders were determined by author consensus as variables likely to be associated with the exposure (beliefs about imaging) and the outcome (imaging referral), and comprised: age, sex, LBP intensity, LBP duration, LBP disability, previous imaging for LBP, previous episodes of LBP, participant diagnosis, and practitioner imaging frequency. Pain duration (4-weeks or less/more than 4-weeks), previous episodes of LBP (yes/no), and participant diagnosis (suspicion of serious pathology/no suspicion of serious pathology) were dichotomised for analysis. Practitioner imaging frequency was calculated from the frequency with which practitioners ordered imaging for patients within the ChiCo cohort. A new item was created with four categories selected to reflect a range of lower to higher frequency of practitioner imaging referral as seen in the literature [1]: less than 15% patients referred for imaging, 15–25%, 26–40%, or more than 40%. The presence of leg pain (no leg pain/leg pain without neurological symptoms/leg pain with neurological symptoms) was added as a potential confounder after the initial analysis. The exposure variables included participant beliefs about the importance of imaging and whether patients wanted imaging.

Correlation between the two imaging beliefs questions and whether patients wanted imaging were calculated using SPSS (IBM Corp. Released 2017. IBM SPSS Statistics for Windows, Version 25.0. Armonk, NY: IBM Corp.), with strong correlation between the two imaging beliefs questions (r = 0.7) and weak correlation between either of the imaging beliefs questions and the question on whether patients wanted imaging (r = 0.3 for each). As the imaging beliefs questions were highly correlated, the scores for the two items were summed to create a single imaging beliefs item on a 2–10 point continuous scale. The single summed beliefs item was then dichotomised for analysis into strong beliefs of the importance of imaging (score of 8–10, where imaging belief questions were either answered with a combination of agree or strongly agree on both questions, or strongly agree on one question and neutral agreement on the other) or uncertain beliefs about the importance of imaging (score of 2–7). Any participants with missing data for either of the imaging beliefs questions were removed from the dataset before the items were summed. Two models were created, one using the summed and dichotomised imaging belief questions, and the other the using the single (Y/N) question on whether participants wanted imaging as independent exposure variables respectively. Odds ratios (OR) and 95% confidence intervals (95%CI) were calculated for the exposure variable in each model. The dichotomised measure for imaging beliefs was selected for analysis to aid interpretation of results. Sensitivity analysis was performed using the summed imaging beliefs measure as a continuous variable to assess for consistency of results. Multicollinearity of the exposure and confounder variables was assessed, and any variable with a VIF of three or more was removed from the models.

## Results

### Participant characteristics

Participant characteristics are presented in Table 1. The majority of participants who answered the questionnaires were male (59.2%), had experienced the current episode of LBP for 4 weeks or less (70.8%), and had experienced previous episodes of LBP (83.2%). One in five patients (21.7%) were referred for imaging (at the first consultation).

**Table 1:** Baseline characteristics of participants within the ChiCo cohort (N = 2818)

Female (n/N, %)	1151/2818 (40.8)
Age, years (mean, SD)	44.5 (13.7)
Education level (n/N, %)	
High school or less	311/2008 (15.5)
Vocational training	590/2008 (29.4)
University education	1033/2008 (51.4)
Other (undefined)	74/2008 (3.7)
Back Beliefs Questionnaire (BBQ)^a^, /45 (mean, SD)	32.3 (5.9)
Low back pain intensity, /10 (mean, SD)	6.7 (2.1)
Pain duration (n/N, %)	
4 weeks or less	1994/2818 (70.8)
More than 4 weeks	824/2818 (29.2)
Low back disability, /100 (mean, SD)	54.9 (23.9)
Previous episodes (n/N, %)	
None	330/1971 (16.7)
1–3	732/1971 (37.1)
More than 3	909/1971 (46.1)
Leg pain	
No leg pain	2000/2818 (71.0)
Leg pain without neurologic symptoms	653/2818 (23.2)
Leg pain with neurologic symptoms	165/2818 (5.9)
Clinical suspicion of serious pathology (n/N, %)	44/2818 (1.6)
Previous low back imaging (n/N, %)	1083/2818 (38.4)
Referred for imaging (n/N, %)	612/2818 (21.7)

SD, standard deviation

^a^BBQ measured out of 45, lower scores indicate more negative beliefs about low back pain

### Participant beliefs about imaging and whether they wanted to receive imaging (Aim 1)

**Fig. 1:**
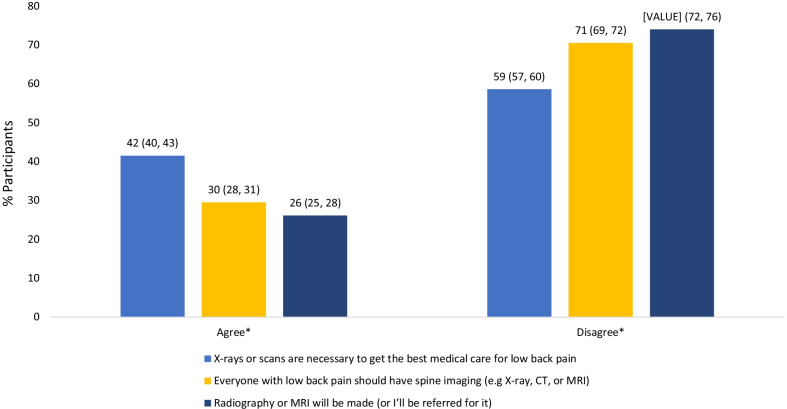
Participant beliefs regarding the importance of imaging for low back pain and desire for referral for imaging at the initial consult; Dichotomised responses. Percentage and 95% confidence interval reported above each column. *Agree indicates selection of ‘strongly agree’ or ‘agree’ for the two imaging beliefs questions or ‘yes’ for whether the participant wanted to receive imaging. Disagree indicates selection of ‘neutral’, ‘disagree’, or ‘strongly disagree’ for the two imaging beliefs questions or ‘no’ for whether the participant wanted to receive imaging. See Additional file 2 for the number of respondents per question and the proportion of respondents selecting each of the Likert scale categories

The dichotomised participant responses are presented in Fig. 1. The number of respondents and Likert scale responses are available in Additional file 2. For imaging beliefs, 41.5% (95%CI 39.6, 43.3) agreed or strongly agreed that ‘X-rays or scans are necessary to get the best medical care for LBP’ and 29.5% (95%CI 27.8, 31.3) agreed or strongly agreed that ‘Everyone with LBP should have spine imaging (e.g. X-ray, CT, or MRI)’. One quarter (26.1%; 95%CI: 24.5, 27.7) of participants wanted to receive imaging of the low back in the initial consult.

### Participant characteristics associated with beliefs regarding the importance of imaging for low back pain and whether participants wanted to receive imaging (Aim 2)

Participants were more likely to believe that imaging was important in the management of LBP and to want an imaging referral at the initial consult if they had a longer duration of LBP, history of previous imaging for LBP, and a lower level of education. Similar associations between participant characteristics and imaging beliefs were seen for both of the two questions about imaging beliefs. Female participants were less likely to believe that imaging was important in the management of LBP, but more likely to want an imaging referral at the initial consult (Table 2).

**Table 2:** Association between participant characteristics and (1) beliefs regarding the importance of imaging for low back pain; and (2) desire for an imaging referral in the ChiCo cohort

	Univariable analysis Odds ratio (95%CI)	Multivariable analysis Odds ratio (95%CI)
*Model 1: X-rays or scans are necessary to get the best medical care for low back pain*^a^		
Sex (female)	0.8 (0.7, 0.9)	0.8 (0.7, 0.9)
Age	1.0 (1.0, 1.0)	1.0 (1.0, 1.0)
Low back pain intensity	1.0 (1.0, 1.1)	1.0 (1.0, 1.0)
Low back pain duration	1.7 (1.5, 2.0)	2.0 (1.7, 2.5)
Back beliefs score	0.9 (0.9, 0.9)	0.9 (0.9, 0.9)
Previous imaging	3.4 (2.9, 4.0)	3.2 (2.7, 3.8)
Low back pain disability	1.0 (1.0, 1.0)	1.0 (1.0, 1.0)
Previous episode of low back pain	1.4 (1.1, 1.7)	1.0 (0.8, 1.3)
Highest completed education (University)	0.8 (0.7, 0.9)	0.8 (0.7, 1.0)
*Model 2: Everyone with low back pain should have spine imaging (e.g. X-ray, CT, or MRI)*^a^		
Sex (female)	0.8 (0.7, 1.0)	0.8 (0.7, 1.0)
Age	1.0 (1.0, 1.0)	1.0 (1.0, 1.0)
Low back pain intensity	1.1 (1.0, 1.1)	1.0 (1.0, 1.1)
Low back pain duration	1.8 (1.5, 2.2)	2.2 (1.8, 2.7)
Back beliefs score	0.9 (0.9, 0.9)	1.0 (1.0, 1.0)
Previous imaging	2.6 (2.2, 3.1)	2.4 (2.0, 2.9)
Low back pain disability	1.0 (1.0, 1.0)	1.0 (1.0, 1.0)
Previous episode of low back pain	1.6 (1.3, 2.0)	1.3 (1.0, 1.6)
Highest completed education (University)	0.8 (0.7, 0.9)	0.8 (0.7, 1.0)
*Model 3: Radiography or MRI will be performed (or I’ll be referred for it)*^a^		
Sex (female)	1.3 (1.1, 1.6)	1.3 (1.1, 1.6)
Age	1.0 (1.0, 1.0)	1.0 (1.0, 1.0)
Low back pain intensity	1.1 (1.0, 1.1)	1.0 (1.0, 1.1)
Low back pain duration	1.9 (1.6, 2.3)	2.2 (1.8, 2.6)
Back beliefs score	1.0 (0.9, 1.0)	1.0 (1.0, 1.0)
Previous imaging	1.3 (1.1, 1.6)	1.2 (1.0, 1.4)
Low back pain disability	1.0 (1.0, 1.0)	1.0 (1.0, 1.0)
Previous episode of low back pain	1.0 (0.9, 1.3)	1.0 (0.7, 1.2)
Highest completed education (University)	0.7 (0.6, 0.9)	0.7 (0.6, 0.8)

Baseline variables: age (continuous variable); sex (dichotomous variable, Male used as reference); LBP intensity (continuous variable, 0–10 scale); low back disability (continuous variable, 0–100 scale), duration of current episode of LBP (dichotomous variable, Less than 4 weeks used as reference); previous imaging for back pain (dichotomous variable, No imaging used as reference), back beliefs (continuous variable, 9–45 scale); education level (dichotomous variable, Below further education used as reference); previous LBP (dichotomous variable, No previous episodes used as reference)

95%CI, 95% confidence interval

^a^Dichotomous variables, higher odds associated with stronger beliefs or desire for imaging

### The association between participant beliefs of the importance of imaging and whether participants wanted to receive imaging, with imaging referral decisions at the initial consult (Aim 3)

Participants who believed that imaging was important in the management of LBP, or wanted imaging at the initial consult, were more likely to be referred for imaging. When adjusted for the pre-selected confounders only whether participants wanted imaging appeared to be independently associated with the decision to refer for imaging (OR; 95%CI 1.6; 1.2, 2.1). Beliefs about the importance of imaging did not appear to be independently associated with imaging referral and the sensitivity analysis using a continuous score produced similar results (Table 3).

**Table 3:** Odds of receiving imaging in participants who (1) believe imaging to be important in the management of low back pain; or (2) want to receive imaging at the initial consult

	Imaging proportion n/N% (95%CI)	Univariable analysis OR (95%CI)	Multivariable analysis OR (95%CI)
*Model 1: Participant beliefs of the importance of imaging for low back pain*			
Beliefs that imaging is important^a^	153/505		
	30.3% (26.5, 34.4)	1.5 (1.2, 1.9)	1.1 (0.8, 1.5)
Beliefs that imaging is not important	340/1,548		
	22.0% (20.0, 24.1)		
Sensitivity analysis: imaging belief questions summedb^b^		1.1 (1.1, 1.2)	1.1 (1.0, 1.1)
*Model 2: Whether participants wanted to receive an imaging referral*			
Wanting to receive imaging^c^	209/550		
	38.0% (34.0, 42.1)	2.5 (2.1, 3.1)	1.6 (1.2, 2.1)
Not wanting to receive imaging	316/1,622		
	19.5% (17.6, 21.5)		

Analysis adjusted for pre-selected confounder variables: age, sex, LBP intensity, LBP duration, LBP disability, previous imaging for LBP, previous episodes of LBP, suspicion of serious pathology, presence of leg pain, and practitioner imaging frequency

OR, odds ratio; 95%CI, 95% confidence interval

^a^Beliefs of the importance of imaging measured as a dichotomous variable. Beliefs that imaging is not important used as the reference group

^b^Summed imaging beliefs measured and assessed as a continuous variable (2–10 point scale). The higher the score, the stronger the belief that imaging is important

^c^Wanting to receive imaging measured as a dichotomous variable. Not wanting to receive imaging used as the reference group

## Discussion

### Main findings

This study found that approximately one third (30%) to two fifths (42%) of patients presenting for chiropractic care for LBP believed that imaging was important for best management. Approximately one quarter (26%) of patients wanted to receive an imaging referral at the initial consult. Patients were more likely to believe in the importance of imaging, or to want an imaging referral, if they had a longer duration of LBP, a history of previous imaging for LBP, and a lower level of education. Whether participants wanted imaging at the initial consult was associated with being more likely to receive an imaging referral, independent of preselected confounders (OR; 95%CI 1.6; 1.2, 2.1), while beliefs about the importance of imaging were not independently associated with receiving an imaging referral.

### Comparison to previous literature

To our knowledge, this is the first study to assess beliefs of the importance of imaging and desire for imaging in patients presenting for chiropractic care. Similar studies have been performed in patients presenting for general medical care [5, 6] and in the general population [7, 8], with a higher percentage of participants believing in the need for imaging, ranging from 48% [6] to 72% [5, 7] than in this study. The differences in imaging beliefs seen in the current study compared to previous studies may result from differences in the clinical setting (chiropractic care compared to medical care), differences in geographic location (Denmark compared to Australia [6], Norway [5, 7], and England [8]), or a change in beliefs over time, with the most recent of the previous studies published in 2016 [6]. The Australian study [6] also assessed the association of baseline characteristics with imaging beliefs and similarly found that history of previous imaging and lower education level were associated with increased beliefs in the need for imaging. However, other variables found to be associated with increased beliefs in the need for imaging in the Australian study [6], including older age and a lower Back Beliefs Questionnaire score, were not consistently associated with beliefs in the current study. Association between beliefs of the need for imaging and imaging referral has been assessed in general medical practice [9], with increased imaging use in patients who thought imaging was necessary. However, the current study is the first to assess participant imaging beliefs and desire for imaging prior to the initial consult, and to assess the impact of imaging beliefs or desire for imaging on subsequent imaging referral in a cohort presenting for chiropractic care.

### Strengths and limitations

The key strengths of this study include the large sample size and prospective data collection. Further strengths are related to the timing of data collection which allowed imaging beliefs and desire for imaging to be assessed prior to the first consultation with the chiropractor, and thus without potential immediate influence from the chiropractor and whether they were referred for imaging during the consult. The timing of data collection, and the consideration of important participant and chiropractor confounders, allowed assessment of whether different participant beliefs, or whether participants wanted imaging, were associated with subsequent imaging referral, without the recognised limitations of previous studies that were performed retrospectively [5, 9].

Limitations are as follows. Generalisability of the findings may be reduced by the limited geographic setting, with participants only recruited from chiropractic practice within a single region in Denmark [17]. Similar studies in other regions may need to be conducted in the future. Data imputation was performed for two covariates (previous LBP and education level) contained within the second baseline survey, with 27% missing responses. Established methods were used to perform the imputation to reduce associated bias [19]. Imputation was considered suitable despite the large percentage of missing responses as these participants did not respond to the second baseline survey in any way, and non-response to these two questions was unlikely to be related to participant choice. All other baseline covariates on which imputation was performed had less than 5% missing data. Education level was not pre-selected as a potential confounder for Aim 3. However, education level was found to be associated with beliefs on the importance of imaging and desire for imaging in Aim 2, and as such may impact the decision to refer for imaging. Post-hoc sensitivity analysis was performed with the addition of education level as a confounder, with no difference in results found (Additional file 3). Finally, although the imaging beliefs questions have been used in previous research [6], they have not been formally evaluated for content validity and comprehension, and misinterpretation of the question by participants may have been possible.

### Implications for clinical practice and research

In the current study, a smaller proportion of participants believed that imaging was important in the management of LBP or wanted to receive imaging in the initial consult when compared to previously published studies. This may imply that participants in the current study were more educated regarding the limited usefulness of imaging in the management of LBP, in line with current evidence [12]. Lower education levels were associated with believing imaging to be important and wanting to receive imaging, and may be a driver of healthcare inequality if not addressed within the healthcare system.

Participants who wanted to receive imaging in the initial consult were more likely to get an imaging referral, highlighting that, although unmeasured variables cannot be excluded, patient desires for imaging may play a role when chiropractors refer for imaging, despite chiropractors reporting that they uncommonly refer for imaging due to patient pressure [14, 15]. The chiropractors in this study were unaware of the questionnaire responses and would not have known if participants wanted imaging unless it was discussed during the initial consult, as would be the case in a standard clinical consult. A patient education intervention [10], which has been shown to be effective in reducing imaging referrals by medical practitioners in Finland [11], may also be of value in chiropractic clinical practice, particularly with patients who want or are requesting an imaging referral that is not aligned with clinical practice guidelines.

Pre-determined variables that were thought likely by the research team to potentially influence the relationship between imaging beliefs, desire for imaging, and subsequent imaging referral were adjusted for in the analysis. The independent association found between whether patients wanted imaging and imaging referral, after adjusting for the pre-selected confounders, suggests a causal association; however, other unmeasured variables that impact imaging referral and were not adjusted for may impact the relationship in an unknown way.

Although approximately 30–40% of participants thought that imaging was important for the management of LBP, only 25% wanted to receive imaging at the initial consult. Interestingly, participants who had received imaging previously were more likely to believe imaging to be important, but were less likely to want to receive imaging; perhaps because this was an investigation that had already been performed. Conversely, females were less likely to believe imaging to be important but more likely to want to receive imaging. These patient perspectives could be explored at an individual level to further understand the relationships between beliefs about the importance of imaging and whether patients want to receive imaging for LBP.

## Conclusion

While less than half of participants who presented to Danish chiropractors believed imaging to be important for the management of LBP, and only one quarter wanted to receive imaging at the initial consult, those who wanted imaging were more likely to receive a subsequent imaging referral. Whether patients want imaging appears to impact the use of diagnostic imaging in Danish chiropractic clinical practice, and patient education resources should be considered when developing interventions to improve the appropriate use of imaging.

## Supplementary Information

**Additional file 1.** STROBE checklist.

**Additional file 2.** Likert-scale responses for participant beliefs regarding the importance of imaging for low back pain and whether participants wanted to receive imaging.

**Additional file 3.** Post-hoc sensitivity analysis.

## Data Availability

ChiCo data are stored and managed at Chiropractic Knowledge Hub (formerly the Nordic Institute of Chiropractic and Clinical Biomechanics) with the University of Southern Denmark (SDU) as the responsible data authority (Danish Data Protection Agency, j.nr.: 2015-57-0008/16–47215). The datasets used and/or analysed during the current study are available from the corresponding author on reasonable request.

## Abbreviations

LBP: Low back pain
OR: Odds ratio
95%CI: 95% Confidence interval
