# Supplementary material for: Patients with low back pain presenting for chiropractic care who want diagnostic imaging are more likely to receive referral for imaging: a cross-sectional study
Source: Chiropr Man Therap. 2022 Apr 4;30:16. doi: 10.1186/s12998-022-00425-5 (PMC8978373; doi:10.1186/s12998-022-00425-5)
Supplement: Supplementary file 2 — Additional file 2. Likert-scale responses for participant beliefs regarding the importance of imaging for low back pain and whether participants wanted to receive imaging. [file 12998_2022_425_MOESM2_ESM.docx]

**Additional file 2: Participant beliefs regarding the importance of imaging for low back pain and whether participants wanted to receive imaging**

|  | **n/N** | **% (95%CI)** |
| --- | --- | --- |
| **X-rays or scans are necessary to get the best medical care for low back pain** | | |
| Strongly agree | 561/2668 | 21.0 (19.5, 22.6) |
| Agree | 545/2668 | 20.4 (18.9, 22.0) |
| Neutral | 1021/2668 | 38.3 (36.4, 40.1) |
| Disagree | 340/2668 | 12.7 (11.5, 14.1) |
| Strongly disagree | 201/2668 | 7.5 (6.6, 8.6) |
|  | | |
| **Everyone with low back pain should receive spinal imaging** | | |
| Strongly agree | 397/2661 | 14.9 (13.6, 16.3) |
| Agree | 389/2661 | 14.6 (13.3, 16.0) |
| Neutral | 1015/2661 | 38.1 (36.3, 40.0) |
| Disagree | 449/2661 | 16.9 (15.5, 18.3) |
| Strongly disagree | 411/2661 | 15.5 (14.1, 16.9) |
|  | | |
| **Radiography or MRI will be performed (or I’ll be referred for it)** | | |
| Yes | 734/2818 | 26.1 (24.5, 27.7) |
| No | 2084/2818 | 74.0 (72.3, 75.5) |

95%CI: 95% confidence interval
