# Supplementary material for: Patients with low back pain presenting for chiropractic care who want diagnostic imaging are more likely to receive referral for imaging: a cross-sectional study
Source: Chiropr Man Therap. 2022 Apr 4;30:16. doi: 10.1186/s12998-022-00425-5 (PMC8978373; doi:10.1186/s12998-022-00425-5)
Supplement: Supplementary file 3 — Additional file 3. Post-hoc sensitivity analysis. [file 12998_2022_425_MOESM3_ESM.docx]

**Additional file 3: Post-hoc sensitivity analysis - Odds of receiving imaging in participants who i) believe imaging to be important in the management of low back pain; or ii) want to receive imaging at the initial consult with additional confounder of educational level**

|  | **Multivariable analysis OR (95%CI)** |
| --- | --- |
| Beliefs that imaging is important | 1.1 (0.9, 1.5) |
| Imaging belief questions summed | 1.1 (1.0, 1.1) |
|  |  |
| Wanting to receive imaging | 1.6 (1.3, 2.1) |

OR: Odds ratio; 95%CI: 95% confidence interval
